# On a Boat: A Case in Australia of Endophthalmitis and Pyogenic Liver, Prostatic, and Lung Abscesses in a Previously Well Patient due to *Klebsiella pneumoniae*

**DOI:** 10.1155/2014/137248

**Published:** 2014-09-17

**Authors:** Alecia Vandevelde, Bojana Stepanovic

**Affiliations:** ^1^Fremantle Hospital, Alma Street, Fremantle, WA 6160, Australia; ^2^Royal Darwin Hospital, Rocklands Drive, Tiwi, NT 0810, Australia

## Abstract

This is a case report about a patient who arrived in our emergency department in Western Australia to the care of the urologists having just gotten off a ship with a bacterial infection that would result in a 44-day stay in hospital and have quite devastating lasting effects for the young male. His story was in fact reflective of an emerging global phenomenon. Once thought to generally be a bacterium of threat only to the elderly and alcoholics, causing pneumonia and urinary tract infections, this case report describes the potentially devastating consequences of what is now becoming recognized as a hypervirulent form of *Klebsiella pneumoniae* with the potential to spread throughout the system rapidly seeding abscesses and causing significant morbidity in nonimmunocompromised patients. Initially noticed in Asia increasingly case reports are emerging in Western countries suggesting a global spread.

## 1. Introduction

Our patient was a 38-year-old male who was originally from Manila and, having spent the last 4 months as a crewman on a ship, would spend the next month and a half in a hospital where the language was not his own and far from all his family and friends. Having departed from Japan, the ship travelled to Tampa via the Panama Canal, arriving in Australian waters months later. He presented to us with a two-week history of fever, back pain, and difficulty passing urine. He spiked temperatures of over 40°C and experienced increasing difficulties with urination, requiring intermittent and finally indwelling, catheter insertion. There were no medical personnel on board and the remarkably adaptable ship's captain under videoconference guidance carried out the catheterisation.

## 2. Case Details

**Figure 1:**
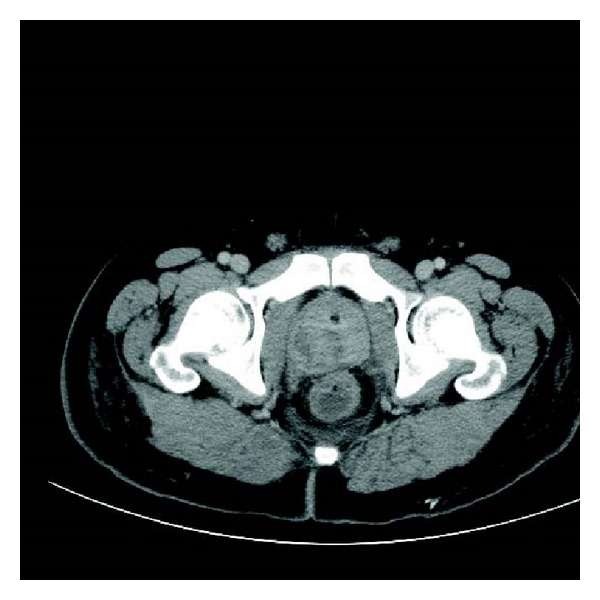
Prostate.

On arrival and initial assessment by the urology team he looked unwell and was understandably distressed and anxious. He explained in broken English that he had begun feeling unwell with lower back pain and fever two weeks earlier. He denied ever having experienced it before. In addition to the difficulties with passing urine he described passing a solid substance per urethra—he was unsure if this was a stone or sediment, and some brief acute scrotal swelling which had largely resolved. He had been given oral ampicillin and tetracycline a few days before the ship's arrival, and when he started experiencing rigors spectinomycin was added. He arrived in our department with an in-out catheter duct taped to his penis. Further questioning revealed no sexual activity during the journey, no history of intravenous drug use, and confirmation that he had been taking antimalarial medication. On first assessment in the emergency department he was initially afebrile with a borderline low blood pressure. He was tachypneic with a respiratory rate of 30 breaths per minute. He had no murmur on auscultation of the heart and had bilateral basal crackles on chest examination. He was tender suprapubically and in the left lower quadrant with no overt flank pain on abdominal exam. Testes were unremarkable to palpation. Per rectal examination revealed smooth and moderately tender prostate, and he had the in-out catheter in place (Figure 1). Requests were made for urgent blood analysis including full blood count, urea and electrolytes, liver function tests, coagulation profile, HIV and hepatitis serology, and malarial films. Urine microscopy, culture, and PCR for sexually transmitted infections were requested as was an urgent CT scan of his renal system including spinal views and chest X-ray. He was clinically very dry and so he was resuscitated with normal saline, and his catheter was replaced. The infectious diseases team on call was consulted and he was commenced on vancomycin and Meropenem at their suggestion. Within half an hour of arrival the patient required urgent review when his oxygen saturation dropped to 85% in room air. He had developed a temperature of 39.5°C and became tachycardic and hypotensive. He received further fluid resuscitation and it was requested that he should have a nurse special admission. His results were discussed urgently with the radiologists.

His abdominal CT showed bilateral collapse and consolidation in both lung bases and left mid zone. Small bilateral pleural effusions were also noted. Within the right lobe of the liver there were two collections seen—one measuring 4.8 × 4.8 mm and the other 3.2 × 3.3 mm, He had marked hydronephrosis of the left kidney with left ureteric dilatation but no calculi seen (Figure 3). A 3 cm prostate abscess was present within the right lobe of the prostate gland. His white cell count was elevated white cell count elevated at 24 × 10^9^, neutrophillia of 20 × 10^9^ CRP elevated at 160 mg/L and his creatinine was 82 umol/L, bilirubin 37 umol/L, ALT 61 U/L, ALP 151 U/L, GGT 84 U/L, and an albumin of 26 g/L, with normal lipase. His lactate was normal. The patient was taken to theatre urgently that night for drainage of his prostatic abscess. Cystoscopy revealed a normal urethra with a prominent bulging right prostatic lobe and a cystitis type picture within the bladder. The right lobe of the prostate was resected down to capsule and expressed with a finger per rectally and a 3-way catheter was inserted for irrigation.

**Figure 2:**
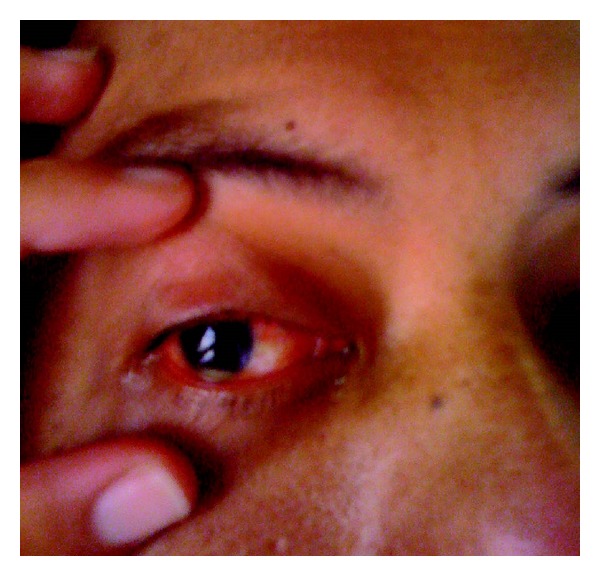
Hypopyon in right eye.

**Figure 3:**
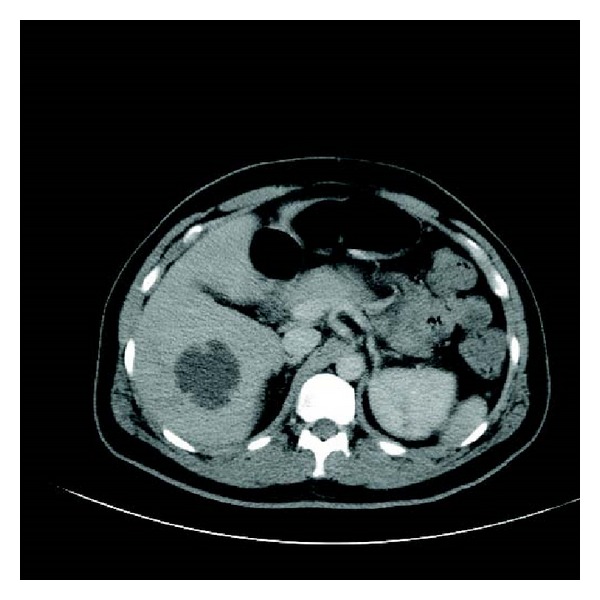
Liver.

The next day the patient mentioned that he was having trouble seeing out of his right eye and a closer look worryingly revealed a hypopyon in his right eye that was clearly there all along but had been missed in the initial assessment (Figure 2). The ophthalmologists were consulted urgently who assessed him as having vision down to hand movements only and a stuck down pupil, with endophthalmitis and retinal detachment. He had an urgent tap of his fluid for culture and injection of antibiotics before being commenced on predniforte, ofloxacin, and homatropine drops with a plan for daily ophthalmology review.

**Figure 4:**
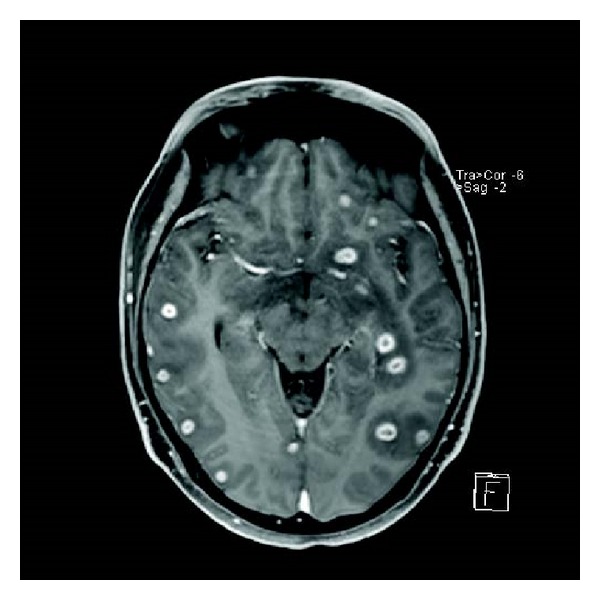
Brain.

Tachypnoea continued to be an issue and the decision was made to also insert a chest drain that drained haemoserous fluid. The patient was at this stage feeling tired but in a stable condition. He continued to spike temperatures greater than 39 degrees celsius and had repeat blood cultures with commencement of chest physiotherapy. Day 2 of admission revealed, as described by the infectious diseases team the “ground breaking news” that he had grown a gram negative on the blood culture and prostatic chips. The vancomycin was therefore ceased and the Meropenem was continued. Ciprofloxacin was started. He was noted to be hypokalemic, which was addressed, with low haemoglobin that was monitored. At this point he was draining 100 mLs of urine per hour. We removed his chest drain on day 3, as the output was minimal. His urine output at this point was 150 mL per hour. During the course of this 3rd day he developed fever in excess of 40°C and began rigoring, with a tachycardia of 180 bpm. He became quite anxious and was reporting chest pain. He was given a small dose of morphine and diazepam and promptly dropped his GCS. A code blue was called where he was given fluids and given a dose of Gentamicin. The gram negative was identified as* klebsiella pneumoniae*. He proceeded to have an urgent CT head and repeat chest and abdomen CT. The CT brain showed no evidence of septic emboli or sinus thrombosis, there was no recollection of his prostatic abscess, and there had been a decrease in the degree of left sided hydronephrosis and the size of the left sided pleural effusion (Figure 4). The appearance of the liver abscess was unchanged.

Despite further administration of normal saline he was persistently hypotensive with a blood pressure of 80/45. Gelofusion was administered and the patient stabilized. Fluid balance was noted at this point to +2020 mL, −975 mL, −965 mL, and then +100 mL after resuscitation.

Another emergency call was made the following day when he again spiked temperatures of greater than 40°C,  and he experienced prolonged rigoring with a supraventricular tachycardia again around 180 bpm. The patient at this stage was shifted to the intensive care unit so he could have invasive monitoring. Given the continued deterioration the decision was made to drain the liver abscess that had initially been managed conservatively. Via percutaneous drainage 45 mLs of frank pus was removed. The drain was not left in situ. He had a urine output of 2800 mL throughout the day. He was returned to the ward on day 5 of admission. Further assessment was carried out at this stage by the ophthalmologists who noted unfortunately an increase in the size of the hypopyon, an increase in injection of the sclera, and an increase in corneal haze. His main complaint was severe right eye pain and continued deterioration in the vision from this eye. Given the poor prognosis it was decided not to undertake removal of vitreous and retinal surgery. Enucleation was deemed too invasive at this point and perseverance with antibiotics was judged the best course of action.

Throughout the course of the day he continued to have a high output of 180–350 mL per hour. His liver function continued to worsen and his albumin was only 28 g/L; so he was switched from Meropenem to Cetriaxone. All electrolyte abnormalities were addressed and he was shifted to a monitored ward. It was uncertain at this time whether his high urine output was representative of a postobstructive state or simply a diuresis because of high input to meet the perceived losses, so after discussion with the medical team on call it was decided to have a trial of stopping his intravenous fluid replacements but to continue with a strict fluid balance and blood pressure monitoring.

By day 6 of admission the results so far included two out of the eight blood culture sets returning positive for* Klebsiella pneumoniae*, sensitive to ciprofloxacin, ceftriaxone, and ampicillin.* Klebsiella* had also been isolated in his prostatic chips, sputum, and vitreous fluid.* Candida* was also noted in his sputum sample. All three urine samples however were negative, as was the pleural fluid samples. The malaria/HIV/Hepatitis B/C serology were negative as was the STI screening. His condition remained unchanged over the next few days with eye pain being his main concern. He was extremely tired and quite flat in affect and described feeling lonely and scared. He had been in contact with his family and ship's insurance company but was understandably extremely worried about his vision. Given the lack of improvement on day 9 a chest tube was inserted on the right side that stayed in place for the next two days. The patient was transferred from the care of the urology team to the medical team.

On day 12 the patient was concerned with increasing amounts of eye pain and alarmingly he was noted by the ophthalmologists to have developed proptosis; so an urgent CT was undertaken. It revealed a concerning progression of his infectious burden with a deterioration in the condition of his right eye that now displayed an increased amount of stranding preseptally and within the vitreous. The collections around the right globe had increased in size. The left eye was fortunately still spared however concerningly the CT now showed multiple enhancing lesions throughout the cortex and cerebellum. All measured approximately 1 cm in diameter and some had central areas of decreased enhancement. Oedematous changes were noted to be surrounding the lesions. Clinical findings up until and at this point were of an encephalopathic nature without any focal neurological findings. These intracranial lesions were discussed with the neurosurgeons, who suggested that there was no drainable collection and that antibiotic treatment should persist. He was started on dexamethasone, the Meropenem recommenced in place of ceftriaxone and the ciprofloxacin dose was increased. It was arranged for the patient to have an MRI, within a week as per the advice of the neurosurgeons, a gallium scan, and a transoesophageal echo (as an earlier transthoracic echo was normal).

The patient's clinical picture finally started to turn around on day 21 of his admission when for the first time he experienced some relief in his eye pain and improvement in ocular movements. The intraocular collection was becoming more organized. Weaning of the dexamethasone was commenced and most of his eye medications were ceased. Surveillance of MRI did not show any progression of his collections. Apart from some increase in discharge from the right eye for which he was given Chloramphenicol ointment and one off low grade temperature of 37.5°C he continued to improve. On day 35 of admission his liver function was noted to be deteriorating again, this time thought most likely to be secondary to Meropenem use. Given the improvement in his clinical picture the Meropenem was ceased. He had a repeat MRI including the abdomen this time that showed a great improvement in the size of all of his collections. He was commenced on oral Cotrimoxazole with the plan to continue it for further six weeks and eventually discharged home to the Philippines only on day 44 after admission.

## 3. Discussion 

Initially described in Asia disseminated bacterial infections in previously fit and well young people secondary to* Klebsiella pneumoniae* are increasingly becoming a global concern [1–4]. The first studies emerged in Taiwan, and in 1986 Lin released a paper for the first time, linking* Klebsiella pneumoniae *with endophthalmitis, where devastatingly all patients lost vision in the infected eye or eyes [5]. Recently however case reports are emerging from all over the world including Saudi Arabia, Ireland, USA, and Australia [1–3].

It has since been discovered that disseminated disease with pyogenic abscesses, particularly pyogenic liver abscesses and endophthalmitis, is most often linked to what is now referred to as hypervirulent serotypes of* Klebsiella pneumoniae* [1, 3–5]. According to a literature review carried out by Siu et al., the lungs, CNS, and eyes are the most common site of spread in those with pyogenic liver abscesses secondary to* Klebsiella pneumoniae* [6]. This type of spread in an immunocompetent host is uncommon of a gram negative [4]. Serotyping of the* Klebsiella pneumoniae* associated with this clinical scenario has identified particular strains. These serotypes (such as K1 and K2) have virulence factors not seen in previous strains and are described as hypervirulent or hypermucoviscous* Klebsiella pneumoniae*. These two particular strains seem to more efficiently increase their capsule production resulting in decreased susceptibility to phagocytosis and neutrophil destruction. Increased biofilm production is thought to be a characteristic, as is the increased ability to capture iron [4]. Currently the most popular idea within the literature is that the port of entry is through the gastrointestinal tract. This is based on studies demonstrating the carriage of these particular strains of* Klebsiella *within the gastrointestinal tract of Asian patients [4].

Initially only seen to cause significant problems in the elderly and immunocompromised patients, such as alcoholics, and these being most commonly pneumonia or urinary tract infections, the bacteria are now increasingly being reported in numerous case reports and studies showing severe invasive infections in young healthy patients [4]. Shon et al.'s review on the topic suggests a mortality rate between 3 and 42% with survivors often left with significant morbidity [4]. Other recent review articles on the topic show patients with disseminated infection to present most often with fever, septicemia, endophthalmitis, meningitis, and genitourinary infections [6]. The predominant symptoms are fever, chills, and pain. Patients are predominantly male and of Asian descent, and there is a significant association with diabetes mellitus [4, 7]. Our unfortunate patient's story could perhaps now be described as an almost classical presentation of a still rare condition. He is male and of Asian dissent, and although not diabetic, who had a particularly widely disseminated infection with positive blood cultures and collections yielding the bacteria in his liver, prostate, and eye. He also had collections in his lung, although sputum was negative for* K. pneumoniae*, and collections intracranially that were not drained but no doubt due to the infection; in fact retrospectively his tiredness was most likely encephalopathic drowsiness secondary to meningitis and developing brain abscesses. All cases so far described in the literature required prolonged courses of antibiotics and our patient did not differ here. Of increasing concern is the identification of carbapenemases which, in combination with extended B-Lactamases, pose major treatment challenges. There is debate about whether percutaneous or operative drainage of liver abscess is the best course of action. It would appear to be reasonable to attempt percutaneous drainage first which, as in our patient, is often successful and less invasive.

Our patient's* Klebsiella* strain was not serotyped as this case occurred when very few cases had been reported and our laboratories did not do this as a routine. This case highlights the importance of early suspicion of this hypervirulent* klebsiella* in those previously fit and well patients presenting unwell with pyogenic abscesses in the liver or elsewhere and the importance of a thorough examination of the entire body so that aggressive management can be initiated early in an infective process that has the potential to run a devastating course.
